# Métastases cutanées d'un carcinome vulvaire

**DOI:** 10.11604/pamj.2015.22.270.6833

**Published:** 2015-11-20

**Authors:** Hanane Rachadi, Inssaf Ramli

**Affiliations:** 1 Service de Dermatologie, Vénérologie, CHU Ibn Sina, Rabat, Maroc

**Keywords:** Métastases cutanées, carcinome épidermoide, cancer vulvaire, Skin Metastases, squamous cell carcinoma, vulvar cancer

## Image en medicine

Les métastases cutanées (MC) sont rares. Elles représentent généralement un stade évolutif avancé de la maladie cancéreuse. BF, âgée de 64 ans, a été suivie pour un carcinome épidermoïde vulvaire traité par vulvectomie totale, curage ganglionnaire bilatéral, radiothérapie externe et chimiothérapie. 6 ans après le traitement, la patiente s'est présentée avec de multiples nodules rouges, fermes, au niveau de l'abdomen et des membres inférieurs. La biopsie des lésions cutanées a été en faveur d'un carcinome épidermoïde. L'origine des lésions cutanées a été attribuée à la néoplasie vulvaire. Le traitement a consisté en une chimiothérapie type CHOP. La patiente est décédée dans un tableau de métastases cérébrales 9 mois après le diagnostic. A la différence des métastases viscérales, lymphonodales ou osseuses, Les MC sont peu fréquentes (0,7 et 9% de toutes les métastases) et leur identification présage d'un mauvais pronostic. Elles peuvent être précoces et parfois révélatrices, concomitantes ou tardives. Elles se présentent le plus souvent comme des nodules cutanés, parfois comme des lésions inflammatoires, cicatricielles ou bulleuses et rarement comme un granulome annulaire ou un ulcère. Les MC peuvent soit reproduire l'aspect histologique de la tumeur primitive, soit former d'autres contingents, parfois plus indifférenciés. Chez la femme, le cancer de sein représente l’étiologie la plus fréquente, suivi du colon, du mélanome, des ovaires et des poumons. Quant au cancer vulvaire, il est rarement métastatique. Les MC sont de mauvais pronostic, près de la moitié des malades décèdent dans les 6 mois suivant le diagnostic.

**Figure 1:**
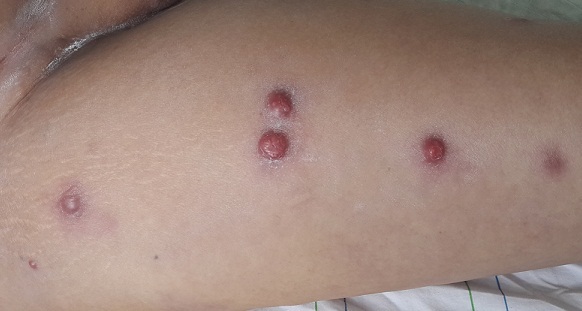
Lésions nodulaires érythémateuses de la cuisse droite

